# Economic and Social Interactions in Business Students during COVID-19 Confinement: Relationship with Sleep Disturbance

**DOI:** 10.3390/bs12040100

**Published:** 2022-04-09

**Authors:** Inmaculada Jimeno García, Anne Marie Garvey

**Affiliations:** Social Dimension and COVID-19, Interdisciplinary Research, Department of Economics and Management, Universidad de Alcalá, 28802 Madrid, Spain; inmaculada.jimeno@uah.es

**Keywords:** sleep, EQLS, students, mental health, quarantine, lockdown, income, family satisfaction, death, infection

## Abstract

The objective of the study was to observe the effect of sleep on students in the first week (Point 1) of strict confinement and to analyze its evolution after one and two months (Point 2) of strict confinement during COVID-19. The evolution of sleep, in association with affected income status, family relations, and the death or infection of a family member or friend by COVID-19 was examined. Students responded to a survey that included questions relating to sleep quality and general well-being from the European Quality of Life Survey (EQLS). Sleep disturbance increased over strict confinement but was substantially more and with greater intensity in the case of students not living with family members. The results show that loss of family income, loss of a family member or friend, and having a friend or family member infected affected sleep quality negatively at Point 1. However, at Point 2, confinement itself affected students sleep, as well as the variables analyzed. Domestic harmony was a positive correlation for better sleep health. The study is important for managing coping policies and diagnosis.

## 1. Introduction

The study examines the effect of confinement and prolonged confinement on sleep during COVID-19. Our research takes a group of business students and contrasts the perceptual effect of quarantine on their sleep. It then contrasts this information with the effect on family income, interactions with family members, and having a friend or family member who was infected by or who died from the virus. The objective of this study is to add to the literature on sleep research and to gain knowledge on the effect that prolonged and strict quarantine measures have on an individual’s sleep pattern and more specifically the effect on sleep when contrasted with other social and economic issues. Students from a business faculty in Madrid disclosed their situation at week one (Point 1) and between weeks four and eight (Point 2) after the enforcement of the confinement measures. The procedure was carried out after a state of emergency was declared in Spain on 14 March 2020 [1] due to the spread of the SARS COVID-19 virus among the Spanish population.

The virus was first detected in Wuhan city in East China (Hubei Province, China) in December 2019 [2]. The highly contagious and infectious virus spread hastily throughout China and cases began to be detected in other countries. The severity of the circumstances caused the World Health Organization (WHO) to declare a global epidemic in the second week of March 2020 [3]. In Spain, a national state of alarm was declared by the Spanish government on 14 March 2020 through the publication of the Real Decreto [1]. On 16 March 2020, a population lockdown began. Universities officially began online learning on this date, after being progressively closed during the previous week (starting from 9 March 2020).

Students in Spain are an interesting sample to study, as Spain was one of the most severely affected countries by the initial quarantine measures taken by a government. The confinement period lasted three months and six days [4], from 16 March until the 21 June. The severe conditions of the confinement imposed included the prohibition of leaving one’s home unless the reasons covered the acquisition of basic necessities including food and pharmaceuticals or similar [1]. These strict lockdown measures were lifted little by little from 2 May 2020.

Sleep problems due to the COVID-19 pandemic and quarantine procedures were predicted [5]. University students were considered to be a highly vulnerable group. One research study [6] examined 226 relevant publications on COVID-19 and sleep disorders up to 30 April 2021 and found that insomnia and sleep disorders were frequently reported, and associations were found with confinement, physical exercise, and social interactions, among others. Another study found that one in four health workers had sleep problems during the outbreak [7]. A high prevalence of sleep problems was found among adolescents and young adults during the COVID-19 epidemic [8,9] but there are few studies to date on university students. Those who suffered uncertainty regarding a possible COVID-19 infection and who had greater fear of direct contact with those infected by COVID-19 had an increased risk of developing sleep disturbances [10]. Another study observed that the effect of confinement during COVID-19 on sleep was greater on students than on workers [11].

A publication on sleep [12] found that more than a third of participants reported increased sleep disturbances during the pandemic. It also highlighted the following issues, that a stricter level of quarantine and pandemic-related factors, for example, financial strain, were associated with poorer sleep health, that domestic conflict was the strongest correlate of poorer sleep health, and that poorer sleep health was strongly associated with greater depression and anxiety symptoms. There are previous studies analyzing the effect of anxiety on the variables analyzed here, but few publications on sleep and less on the evolution of sleep. Cao et al. (2020) detected that a negative economic effect, the effects on daily life, and delays in academic activities were positively associated with anxiety symptoms, but they did not analyze sleep problems [13]. High levels of psychological distress on 129 quarantined individuals were discovered some years ago [14]. A research team found that when emotional well-being (quality of sleep or death of a relative) is reduced and material well-being is negatively affected (income level), anxiety levels are increased [15]. Rezaei et al. (2021) carried out one of the largest and most representative analyses of sleep [16] and detected alterations in sleep patterns and heart rate during confinement with a return to normality after confinement. Petrov et al. (2021) detected a negative correlation on sleep quality depending on the severity of change in routines, and family stress and discord was detected [17]. In order to mitigate the problems, governments need to be prepared to maintain health and care spending in line with demand [18], and viruses and health systems should not discriminate and should give the same health rights to all, regardless of their income level [19].

In the next section, the objectives and methods of the study are examined, in Section 3 the results and discussion are presented, and Section 4 covers the conclusion.

## 2. Materials and Methods

The target population were undergraduate students at the Business, Economics, and Tourism Faculty at Alcalá University in Madrid. At Point 1, 195 students answered the survey and 135 students participated in both surveys (see Appendix A), which represented 69.23% of the total population. Online consent was given by students before participation, and they were also informed of the purpose of the study.

The survey was prepared through Microsoft Forms and consisted of 12 questions. The survey was passed at two different moments during strict confinement. The questions included some introductory demography questions on gender, academic information, and living arrangements, as well as a series of questions relating to people’s quality of life including sleep quality and how the virus was affecting personal emotions. Summarizing, the survey consisted of three areas: (1) demographic data; (2) sleep disturbance; and (3) family income, level of family satisfaction, and the death or infection of family members or friends by the virus. Demographic data were collected on gender, academic information, and living arrangements.

The quality-of-life survey was structured in line with the definition by Verdugo et al. [20]. These authors state that the quality of life of a person is composed of eight dimensions (emotional well-being, interpersonal relationships, material well-being, personal development, physical well-being, self-determination, social inclusion, and rights). This study analyzed the relationship that some of these dimensions have with sleep quality during the pandemic.

The well-being and quality of life questions used were taken from the European Quality of Life Survey (EQLS). It is a cross-sectional survey carried out by the European Foundation. A descriptive statistics analysis was carried out to explain the demographic and other questions at two different moments of time. A univariant analysis was used to determine if there were any univariate associations between sleep disturbance and family income, interactions with family members, and having a friend or family member infected or who died from the virus. The statistical analysis was performed using SPSS Version 26.

## 3. Results

The demographic and selected characteristics of the study population are included in Table 1. It can be observed that of the total sample, 70.4% were female and the remaining 29.6% were male. All were studying a business-related degree, 54.81% were second year students and 93.3% of students were living with their family during the confinement period. A dependent relation was found in the case of income being negatively affected at Point 1, but not at Point 2. For the other variables, a dependent relation was not considered a feasible measurement due to the numbers being disproportionate at those early points. We consider that the results are useful and insightful however and are an important advance in sleep literature and on the effects of prolonged confinement.

### 3.1. Relation between Disturbed Sleep, Living with Family, and Degree of Satisfaction with Family during Strict Confinement

Firstly, we examined the relation between disturbed sleep during COVID-19 and living with family (Table 2). We also examined the relation between disturbed sleep and the perception of feeling satisfied or not with family (Table 2). It is important to mention that the proportion of students living with family was much higher than those living alone or with non-family members (93.3% vs. 6.7%, respectively, at Point 1 and 94.8% vs. 5.2%, respectively, at Point 2).

We observed that a similar proportion of students living and not living with family members (23.8% and 22.2%, respectively) were not affected by disturbed sleep at Point 1. However, this percentage was reduced both in students who lived with their families and those who lived alone or with non-family members (14.9% and 0%, respectively) at Point 2. In other words, the proportion of students who had problems managing sleep increased as time passed, and it can be said that it was higher in those students who do not live with their respective families (Table 2).

At Point 1, we observed that 41.26% of students who lived with their families had sleep problems more than half of the time to all of the time in comparison with 33.3% of those who did not live with family members. This situation reversed over time; at Point 2 we observed that 85.7% of students who did not live with their families recognized having problems with the quality and management of sleep more than half of the time to all of the time. This was compared to 60.09% in the case of students living with their families. We conclude therefore that sleep disturbance increased for all students from Point 1 to Point 2, but substantially more and with greater intensity in the case of students not living with family members (Table 2).

A small group of students perceived dissatisfaction with their family at Point 1 (3.7%) and Point 2 (13.5%), and we observe that 60% of them said they slept worse all the time at Points 1 and 2 (Table 2). This indicates severe levels of sleep disturbance among students who perceived being dissatisfied with their family. There was more perceived dissatisfaction with family at Point 2.

We observed that the proportion of students who were not affected by disturbed sleep at Point 1 and 2 was higher among those who were very satisfied with their families (42.1% at Point 1 and 43.7% at Point 2) or completely satisfied with their families (36.8% at Point 1 and 37.5% at Point 2). This indicates that family satisfaction is important in relation to not suffering sleep disturbance.

In the total sample, students that slept worse all the time at Point 1, 25% were completely satisfied with the family and 40% were very satisfied. However, these percentages changed their distribution when we asked students again at Point 2, showing 33.3% that were completely satisfied and another 33.3% who were very satisfied with family. The sum of these satisfaction levels was similar at both points, but there was an increase in the completely satisfied level and a reduction in the very satisfied classification.

### 3.2. Relation between Disturbed Sleep and the Negative Effect on the Immediate Family Income (Parents, Siblings)

We contrasted the variable sleep and the impact on economic income (Table 3), and this contrast showed a dependent relationship at Point 1. However, when we tested both variables again at Point 2, it appeared that there was no significant dependent relation between both variables. This may be because individuals were more worried about their health or confinement affected them more than the effect on the family income at Point 2. This may also be because of the government’s aid to those whose income level was impacted.

At Point 1, if the family’s income was negatively affected, the quality of the students’ sleep worsened. At this point. 46.7% of the students recognized that their family income has been affected. This percentage increased to 63% at Point 2. When we examined more closely the students from these affected families, we found that 11.1% at Point 1 and 15.3% at Point 2 did not suffer from disturbed sleep. However, the data also show that a high proportion of the students from the affected families suffered from disturbed sleep more than half of the time to all of the time (47.6% at Point 1 and 64.7% at Point 2). In students from families whose income was not negatively impacted, we observed that 34.7% of them did not show any sleep problems at Point 1. However, this percentage reduced to 12.2% at Point 2. Sleep disturbance increased after prolonged and strict confinement even in the case of students whose families did not suffer from a loss of income. In fact, at Point 2, 59.2% of these students showed sleep problems more than half of the time to all of the time, in comparison with 64.7% of students from families with income loss. We conclude that something more than income loss affected students’ sleep during the prolonged confinement period. The data indicate that at Point 2, despite a diminished quality of life of students whose family income was affected, this factor lost relevance as confinement was prolonged.

### 3.3. Relation between Disturbed Sleep and the Death of a Family Member or Friend

At Point 1, 80% of the students who experienced the death of a family member showed some type of sleep disturbance (see Table 4). In fact, 70% of them admitted to having sleep problems more than half of the time to all of the time. At Point 2, the results were 91.7% and 75%, respectively, showing an increase in sleep disturbance from Point 1 to Point 2. Of the students who did not experience the death of a family member, 76% suffered some sort of sleep disturbance at Point 1 (38.4% most of the time to all of the time). Interestingly, however, at Point 2, the percentages increased to 85.2% and 61.5% respectively. Initially, the loss of a family member to COVID-19 showed higher levels of sleep disturbance at both points.

At Point 1, only 4.4% of students had experienced the death of a friend (Table 4). Of this group, almost 83.3% suffered from disturbed sleep (66.7% more than half to all of the time). At Point 2, just 6.67% of students had suffered the loss of a friend and 88.9% of them suffered some form of sleep disturbance (44.4% more than half of the time to all of the time). The intensity of sleep disturbance dropped dramatically at Point 2, probably due to the fact that 66.7% of the students who suffered the loss of a friend at Point 2 were the same as those who suffered the loss at Point 1, giving time to assimilate the situation. The intensity of sleep disturbance increased at Point 2 in the case of the death of a family member.

### 3.4. Relation between Disturbed Sleep and a Family Member or Friend Infected by COVID-19

It is important to highlight that the proportion of the sample that had an infected family member was 25.92% at Point 1 and 31% at Point 2. At Point 1, we observed that of the students who had an infected family member, 14.3% of them did not suffer from disturbed sleep (see Table 5). However, this percentage decreased to 4.9% at Point 2. When we analyzed the data of those students who did not have an infected family member, 24.5% of them were not affected by sleep disturbance at Point 1 and this dropped to 18.3% at Point 2. We observed therefore, more sleep disturbance in students with a family member infected.

At Point 1, of the students who had an infected family member, 42.8% of them admitted to having sleep problems more than half of the time. This was 45.5% in the case of students who did not have an infected family member. At Point 2, 68.3% of students with a family member infected admitted to having sleep problems more than half of the time to all of the time, and this was 60.2% in students who did not have an infected family member. At Point 1, the intensity of sleep disturbance was higher in those who had no family member infected, and the situation was reversed at Point 2.

Our study continues with the analysis of sleep problems in relation to having a friend infected (Table 5). At Point 1, 36.3% of the sample had an infected friend. This percentage increased to 43% at Point 2. In week one of confinement, the percentage of students who were having problems managing their sleep more than half of the time to all of the time was 51.02%, and it increased to 69.49% at Point 2.

On the other hand, of those who do not have any infected friends, 31.4% had no problem with sleep management at Point 1 and this percentage was 17.10% at Point 2, showing an increase in sleep difficulty over time generally. The numbers corresponding to those with a friend infected and who had no sleep disturbance were 10.2% at Point 1 and 10.16 at Point 2. However, the numbers changed regarding problems managing sleep from more than half of the time to all of the time. In the first week of confinement, 34.9% of students who did not have an infected friend had disturbed sleep more than half of the time to all of the time, but the percentage increased to 56.6% at Point 2. For those with a friend infected, 51% expressed suffering from sleep disturbance at Point 1 and this increased to 69.4% at Point 2. For those students with a friend infected, the intensity of sleep disturbance was higher than that for students with no friend infected. The main results of the research are shown in Table 6.

## 4. Discussion and Conclusions

This study confirms that prolonged confinement and, in this case, strict confinement, have a significant negative impact on sleep disturbance on students. The study is an important advance on the effects of prolonged and strict quarantine on sleep quality and the negative impact that affected income level, dissatisfied family relations, and death and infection of a family member or friend have on sleep quality. At Point 2, it is observed that confinement itself or other factors may be affecting sleep disturbance more than the variables analyzed, which could indicate that confinement in itself and especially strict confinement in this case has negative effects on individuals. A larger sample size would have given more accurate results and the inclusion of further questions in the survey in relation to whether the students had access to a garden, balcony, or open-air during confinement, as well as another question on their physical condition and lifestyle would have added to findings as to whether these were protective factors for sleep health. The above can be considered limitations to the study. Future areas of study at this point would be to focus on the continuing effects on sleep disturbance after the confinement period, to identify those sections of the population that continue to have problems and to identify the reasons and at the same time examine the measures taken by individuals to mitigate both sleep and anxiety problems. The number of individuals seeking mental health treatment presently has increased as a result of the effects of the pandemic. Government aid to those whose income level is affected can decrease the effects on sleep disturbance, and also family support contributes to the mitigation of these negative effects, showing that policies to support families is very important for the general well-being of the population. The observations are important for taking public health decisions and for implementing procedures by authorities in education and other establishments to prevent the negative effects of quarantine on students. A mandatory plan should be implemented by these organizations for crisis situations to mitigate the negative effects on the mental health of students.

## Figures and Tables

**Table 1:** Demographic and living characteristics.

Characteristic	Number	% Mean (SD)
Age (mean = 20)	135	33.8 (3.61)
**Gender**		
Male	40	29.6
Female	95	70.4
**Studies**		
Business management	21	15.6
Law and business	29	21.5
Accounting and finance	23	17
Tourism and business	7	5.2
Economics	9	6.7
Economics and international business	43	31.9
**Course**		
Second	74	54.81
Third	45	33.33
Fourth	16	11.85
**Living arrangements**		
With parents (Point 1)	126	93.3
Alone or with friends (Point 1)	9	6.7
With parents (Point 2)	128	94.8
Alone or with friends (Point 2)	7	5.2

Source: own.

**Table 2:** Relation between disturbed sleep, living with family, and the degree of satisfaction with family during strict confinement.

**Living with Family during Lockdown**	**Disturbed Sleep**											
	**One Week after Lockdown**						**One Month to Two after Lockdown ^1^**					
	**Never**	**Sometimes**	**Less Than Half the Time**	**More Than Half the Time**	**Most of the Time**	**All the Time**	**Never**	**Sometimes**	**Less Than** **Half the Time**	**More Than Half the Time**	**Most of the Time**	**All the Time**
**Yes**	30	33	11	13	20	19	19	18	12	12	32	34
**No**	2	3	1	0	2	1	0	1	0	3	1	2
**Total**	32	36	12	13	22	20	19	19	12	15	33	36
**Frequency (%)**	23.7	26.7	8.9	9.6	16.3	14.8	14.2	14.2	8.9	11.2	24.6	26.9
**Perception of Feeling Satisfied with Family during Lockdown**	**Disturbed Sleep**											
	**One Week after Lockdown**						**One Month to Two after Lockdown ^1^**					
	**Never**	**Sometimes**	**Less Than Half the Time**	**More Than Half the Time**	**Most of the Time**	**All the Time**	**Never**	**Sometimes**	**Less Than Half the Time**	**More Than Half the Time**	**Most of the Time**	**All the Time**
**Not very satisfied**	0	1	0	1	0	3	1	1	0	1	1	6
**Neither satisfied nor dissatisfied**	2	2	0	0	3	1	0	0	1	2	2	0
**Somewhat satisfied**	4	5	0	2	5	3	3	2	2	3	9	4
**Very satisfied**	14	16	4	7	7	8	8	7	5	3	12	13
**Completely satisfied**	12	12	8	3	7	5	7	9	4	6	9	13
**Total**	32	36	12	13	22	20	19	19	12	15	33	36
**Frequency (%)**	23.7	26.7	8.9	9.6	16.3	14.8	14.2	14.2	8.9	11.2	24.6	26.9

^1^ There is 1 missing value at Point 2. Source: own.

**Table 3:** Disturbed sleep and the negative effect on immediate family income.

Family Income Affected Negatively	Disturbed Sleep											
	One Week after Lockdown						One Month to Two after Lockdown ^1^					
	Never	Sometimes	Less Than Half the Time	More Than Half the Time	Most of the Time	All the Time	Never	Sometimes	Less Than Half the Time	More Than Half the Time	Most of the Time	All the Time
**Yes**	7	19	7	5	10	15	13	7	10	12	18	25
**No**	25	17	5	8	12	5	6	12	2	3	15	11
**Total**	32	36	12	13	22	20	19	19	12	15	33	36
**Frequency (%)**	23.7	26.7	8.9	9.6	16.3	14.8	14.2	14.2	8.9	11.2	24.6	26.9

^1^ There is one missing value at Point 2. Source: own.

**Table 4:** Disturbed sleep and death of a family member/friend.

**Death of a Family Member**	**Disturbed Sleep**											
	**One Week after Lockdown**						**One Month to Two after Lockdown ^1^**					
	**Never**	**Sometimes**	**Less Than Half the Time**	**More Than Half the Time**	**Most of the Time**	**All the Time**	**Never**	**Sometimes**	**Less Than Half the Time**	**More Than Half the Time**	**Most of the Time**	**All the Time**
**Yes**	2	0	1	1	5	1	1	2	0	2	2	5
**No**	30	36	11	12	17	19	18	17	12	13	31	31
**Total**	32	36	12	13	22	20	19	19	12	15	33	36
**Frequency (%)**	23.7	26.7	8.9	9.6	16.3	14.8	14.2	14.2	8.9	11.2	24.6	26.9
**Affected by the Death of a Friend**	**Disturbed Sleep**											
	**One Week after Lockdown**						**One Month to Two after Lockdown ^1^**					
	**Never**	**Sometimes**	**Less Than Half the Time**	**More Than Half the Time**	**Most of the Time**	**All the Time**	**Never**	**Sometimes**	**Less Than Half the Time**	**More Than Half the Time**	**Most of the Time**	**All the Time**
**Yes**	1	1	0	0	1	3	1	3	1	0	0	4
**No**	31	35	12	13	21	17	18	16	11	15	33	32
**Total**	32	36	12	13	22	20	19	19	12	15	33	36
**Frequency (%)**	23.7	26.7	8.9	9.6	16.3	14.8	14.2	14.2	8.9	11.2	24.6	26.9

^1^ There is one missing value at Point 2. Source: own.

**Table 5:** Disturbed sleep and a family member or friend infected by COVID-19.

**Affected by a Family Member Infected by COVID-19**	**Disturbed Sleep**											
	**One Week after Lockdown**						**One Month to Two after Lockdown ^1^**					
	**Never**	**Sometimes**	**Less Than Half the Time**	**More Than Half the Time**	**Most of the Time**	**All the Time**	**Never**	**Sometimes**	**Less Than Half the Time**	**More Than Half the Time**	**Most of the Time**	**All the Time**
**Yes**	5	11	4	6	3	6	2	7	4	7	9	12
**No**	27	25	8	17	19	14	17	12	8	8	24	24
**Total**	32	36	12	13	22	20	19	19	12	15	33	36
**Frequency (%)**	23.7	26.7	8.9	9.6	16.3	14.8	14.2	14.2	8.9	11.2	24.6	26.9
**Affected by a Friend Infected by COVID-19**	**Disturbed Sleep**											
	**One Week after Lockdown**						**One Month to Two after Lockdown ^1^**					
	**Never**	**Sometimes**	**Less Than Half the Time**	**More Than Half the Time**	**Most of the Time**	**All the Time**	**Never**	**Sometimes**	**Less** **Than** **Half the Time**	**More Than Half the Time**	**Most of the Time**	**All the Time**
**Yes**	5	12	7	7	6	12	6	8	3	3	18	20
**No**	27	24	5	6	16	8	13	11	9	12	15	16
**Total**	32	36	12	13	22	20	19	19	12	15	33	36
**Frequency (%)**	23.7	26.7	8.9	9.6	16.3	14.8	14.2	14.2	8.9	11.2	24.6	26.9

^1^ There is one missing value at Point 2. Source: own.

**Table 6:** Main Results.

Main Results Obtained from This Research
Sleep disturbance increases from Point 1 to Point 2.
Sleep disturbance is higher and more intense in students not living with the family at Point 2. The opposite is the case at Point 1.
Severe levels of sleep disturbance in students who perceived being dissatisfied with family at both Points 1 and 2.
Perceived dissatisfaction with family increased at Point 2.
Students who perceived to be satisfied with their family suffered less sleep disturbance at Point 1 and Point 2.
In students who suffered sleep disturbance all the time, the positive perception of the family was similar at Points 1 and 2, but the intensity of satisfaction was higher at Point 2.
Something more than income loss affected students sleep at Point 2. The loss of income factor lost relevance after prolonged confinement.
The death of a family member showed high levels of sleep disturbance at Points 1 and 2, and higher intensity at Point 2.
Students who suffered the death of a friend suffered high levels of sleep disturbance at Point 1 and 2 but the intensity of sleep disturbance was substantially reduced at Point 2 probably because 2/3′ s of them had some time to recover from the death of the friend.
Higher levels of sleep disturbance in students with a family member infected at both Points 1 and 2.
At Point 1, the intensity of sleep disturbance is higher in students with no family member infected. This situation is reversed at Point 2.
Higher intensity of sleep disturbance in students with a friend infected than those with no friend infected at both Points 1 and 2.
Those students who had a friend infected, about 10% had no sleep disturbance at both Points 1 and 2.

## Data Availability

The data presented in this study are available upon request from the corresponding author. The data are not publicly available due to being unnecessary for the understanding of the document.

## Appendix A

**Table A1:** SURVEY at Point 1 and Point 2.

Questions	Possible Answer
1. Age	
2. Gender	Male Female Other
3. Studies	Business management Law and business Accounting and finance Tourism and business Economics Economics and international business
4. Academic course	Second year Third year Fourth year
5. Living arrangements	Living with parents Living alone or with friends
6. I sleep badly	All the time Most of the time More than half of the time Less than half of the time Sometimes Never I do not know
7. How satisfied are you with the relationships you have with your family?	Completely satisfied Very satisfied Somewhat satisfied Neither satisfied nor dissatisfied Not very satisfied Not satisfied at all I do not know
8. During the epidemic, has the total immediate family income (parents, siblings) been negatively affected?	Yes No
9. Has a family member died?	Yes No
10. Has a friend died?	Yes No
11. Has a family member been infected?	Yes No
12. Has a friend been infected?	Yes No
